# Vaccination route can significantly alter the innate lymphoid cell subsets: a feedback between IL-13 and IFN-γ

**DOI:** 10.1038/s41541-018-0048-6

**Published:** 2018-03-12

**Authors:** Zheyi Li, Ronald J. Jackson, Charani Ranasinghe

**Affiliations:** Molecular Mucosal Vaccine Immunology Group, Department of Immunology and Infectious Disease, The John Curtin School of Medical Research (JCSMR), The Australian National University, Canberra, ACT 2601 Australia

## Abstract

This study demonstrates that the fate of a vaccine is influenced by the cytokines produced by the innate lymphoid cells (ILC) recruited to the vaccination site, and it is vaccine route and adjuvant dependent. Intranasal virus vaccination induced ST2/IL-33R^+^ ILC2 in lung, while intramuscular vaccination induced exclusively IL-25R^+^ ILC2 in muscle. Interestingly, a larger proportion of IL-13^+^ ILC2s were detected in muscle following i.m. viral vector vaccination compared to lung post i.n. delivery. These observations revealed that ILC2 were the main source of IL-13 at the vaccination site (24 h post vaccination) responsible for inducing T cells of varying avidities. Moreover, recombinant fowlpox viral vector-based vaccines expressing adjuvants that transiently block IL-13 signalling at the vaccination site using different mechanisms (IL-4R antagonist or IL-13Rα2 adjuvants), revealed that the level of IL-13 present in the milieu also significantly influenced IFN-γ, IL-22 or IL-17A expression by ILC1/ILC3. Specifically, an early IL-13 and IFN-γ co-dependency at the ILC level may also be associated with shaping the downstream antibody responses, supporting the notion that differentially regulating IL-13 signalling via STAT6 or IL-13Rα2 pathways can modify ILC function and the resulting adaptive T- and B-cell immune outcomes reported previously. Moreover, unlike chronic inflammatory or experimentally induced conditions, viral vector vaccination induced uniquely different ILC profiles (i.e., expression of CD127 only on ILC2 not ILC1/ILC3; expression of IFN-γ in both NKP46^+^ and NKp46^−^ ILCs). Collectively, our data highlight that tailoring a vaccine vector/adjuvant to modulate the ILC cytokine profile according to the target pathogen, may help design more efficacious vaccines in the future.

## Vaccine efficacy: Route of administration implicates different protective signalling molecules

Immune-system cells express different protective signalling molecules dependent on the route of vaccine administration and co-administered immune-system stimulators. A group of researchers from the Australian National University, led by Charani Ranasinghe, performed vaccinations on mice either intramuscularly or intranasally, with and without an adjuvant—a chemical administered with vaccines to make them more effective. The team’s results revealed that intranasal vaccination and intramuscular vaccination induce different types of the recently identified innate lymphoid cells (ILCs), and interleukin (IL) signalling molecules. Adjuvants also influence the ILC/IL response. The variation in interleukin signalling can directly influence downstream immune responses such as T-cell activation, and therefore vaccine efficacy. This study suggests that vaccine routes of administration and adjuvants can be tailored to induce IL responses preferable to fighting individual pathogens, therefore creating more effective prophylactics.

## Introduction

Innate lymphoid cells (ILCs) are a recently identified class of immune cells that do not express antigen receptors nor surface markers characteristic of other immune cells, i.e., lineage negative.^1^ ILCs are known to play a multi-factorial role at the mucosae,^2^ for example, in tissue remodelling,^3^ allergy and inflammation,^4,5^ Crohn’s disease,^1^ and immunity towards helminth and intracellular parasitic infections.^4–6^ ILCs are thought to develop from a common lymphoid progenitor,^1,7^ and according the transcription factors and cytokines they express, ILC have been broadly classified into three main categories. ILC1 respond to IL-12, IL-18 and IL-15 and express transcription factor T-bet, interferon (IFN)-γ and tumour necrosis factor (TNF)-α. ILC2 subsets are characterised by surface receptors IL-33R^+^ (ST2^+^), IL-25R^+^ (IL-17RB^+^) or TSLPR^+^ and can be stimulated by IL-33, IL-25 (IL-17E) or thymic stromal lymphopoietin (TSLP), respectively. Activated ILC2 express GATA3, IL-13, IL-5, IL-9 or IL-4. In contrast ILC3 respond to IL-1β and IL-23 and express RORγt, IL-22 and IL-17A.^1,7,8^ However, recent studies indicate strong plasticity between ILC2, ILC1 and ILC3 subsets according to the tissue environment and the external stimuli they encounter.^9–11^

Studies have shown that influenza virus^4,12^ and rhinovirus infection stimulate ILC2 IL-13 expression and exacerbate asthma responses,^13^ and HIV infection causes an irreversible loss of ILC function during acute infection.^14^ However, how different ILC subsets are modulated during viral infection or vaccination and influence vaccine-specific immunity is poorly understood. A range of recombinant viruses, including Avipoxviruses (canarypox and fowlpox viruses) used in the HIV RV144 Thai trial,^15^ Modified Vaccinia Ankara (MVA) and Adenovirus-5 are being developed as vectors to deliver vaccines for human diseases, However, the mechanisms by which these different vaccine vectors modulate innate immunity is not fully understood. MVA is known to stimulate TLR2, TLR6 and NALP3 inflammasome pathways, with vigorous IFN-β and IL-1β expression by macrophages.^16^ While ILC2 IL-13 expression licences CD11b^+^ CD103^−^ conventional dendritic cells to stimulate CD4^+^ T-helper 2 (Th2) responses.^17^ However, the influence of ILC interacting with professional antigen-presenting cells (APCs) in stimulating of antiviral Th1 immunity is not known. Thus, understanding how these vaccine vectors interact with the innate immune response and influence resulting adaptive immunity is paramount for developing efficacious vaccine technologies in the future.

Our previous studies, have revealed that (i) recombinant poxvirus HIV-1 antigen vaccines delivered via the mucosa induce high avidity, poly-functional HIV-specific CD8^+^ T cells with reduced IL-4 and IL-13 expression,^18^ (ii) IL-13Rα2 and IL-4R antagonist-adjuvanted vaccines transiently blocking IL-13 and/or IL-4 signalling at the vaccination site induce higher avidity/multi-functional HIV-specific effector/memory CD8^+^ T cells with improved CD8^+^ T-cell-mediated protective efficacy^19,20^ and (iii) IL-4R antagonist adjuvant vaccine also induces HIV gag-specific IgG1 and IgG2a antibodies.^20^ (The IL-13Rα2-adjuvanted vaccine co-expresses HIV antigens together with soluble IL-13Rα2 and can block IL-13 activity at the vaccination site. Whereas the IL-4R antagonist-adjuvanted vaccine co-expresses HIV antigens and C-terminal deletion mutant of the mouse IL-4 without the essential tyrosine required for signalling, which can bind to both type I and type II IL-4 receptor complexes with high affinity, and transiently block both IL-4 and IL-13 signalling at the vaccination site).^21^ Interestingly, the responses observed with the adjuvanted vaccines in mice were similar to what has been reported for elite controllers who naturally control HIV infection and do not progress to clinical AIDS.^22–24^ While we have gained some understanding of how IL-4/IL-13 regulates CD8^+^ T-cell avidity at the adaptive immune level,^25–27^ it is still unclear which cells in the innate immune compartment are involved in IL-4, IL-13 and IFN-γ expression and/or regulation at the vaccination site, responsible for the downstream T- and B-cell outcomes.

## Results

### Intranasal vaccination induces lineage^−^ ST2/IL-33R^+^ ILC2s-expressing IL-13 at the lung mucosae and IL-4R antagonist/IL-13Rα2-adjuvanted vaccines inhibit this activity

**Fig. 1:**
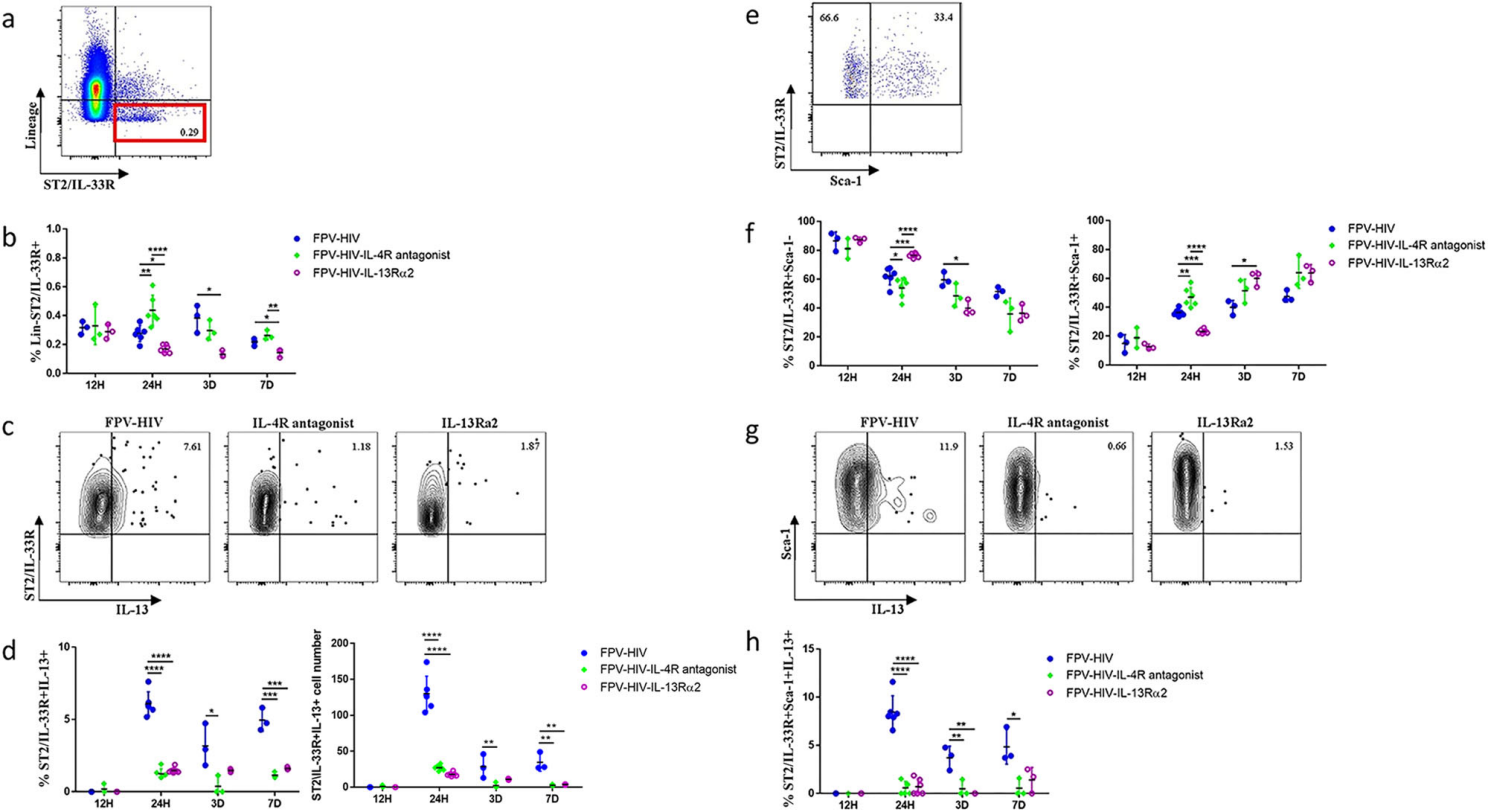
Evaluation of lung ILC2 and IL-13 expression following intranasal vaccination. In this study, ILC2 cells were identified as CD45^+^ FSC^low^ SSC^low^ lineage^−^ST2/IL-33R^+^ cells using flow cytometry (**a**). BALB/c mice were immunised intranasally with FPV-HIV, FPV-HIV-IL-4 antagonist and FPV-HIV-IL-13Rα2 vaccines, and the percentage of lung lineage^−^ ST2/IL-33R^+^ ILC2 (**b**) and their IL-13 expression (**c**, **d**) were assessed at 12 h, 24 h, 3 days, and 7 days post vaccination. The FACS plots (**c**) are representative of the 24 h data points for each vaccination. **d** The left graph represents percentage of ST2/IL-33R^+^ IL-13^+^ cells and right graph the total cell number. The lineage^-^ ST2/IL-33R^+^ cells were further analysed for Sca-1 and IL-13 expression (**e**–**h**). Data indicate that IL-13 was only detected in lineage^-^ ST2/IL-33R^+^ Sca-1^+^ ILC2 while no IL-13 was detected in lineage^-^ ST2/IL-33R^+^ Sca-1^-^ ILC2 (**g**–**h**). The graphs represent the mean and standard deviation (s.d.). The *p*-values were calculated using GraphPad Prism software (version 6.05 for Windows). *p < 0.05, **p < 0.01, ***p < 0.001, ****p < 0.0001. For each time point, experiments were repeated minimum three times. Note that during early time points, no significant inflammatory infiltrates were detected and the absolute cell numbers obtained were very similar

Previous studies in our laboratory have shown that 24 h post intranasal IL-4R antagonist and IL-13Rα2-adjuvanted vaccination can alter IL-4/IL-13 signalling at the vaccination site^19^ and this directly influences the activity of APCs at the lung mucosae resulting in high-avidity CD8^+^ T-cell-mediated immunity.^28^ In this study, we have embarked upon understanding how the expression of IL-4 and/or IL-13 by ILC at the lung mucosae can modulate adaptive immune outcomes following intranasal vaccination using (i) Fowlpox virus (FPV)-HIV unadjuvanted, (ii) FPV-HIV-IL-4R antagonist and (iii) FPV-HIV-IL-13Rα2-adjuvanted vaccines. Firstly, 12 h to 7 days post intranasally (i.n.) vaccination the lung ILC2 were evaluated as described in methods (Fig. 1a). ILC2 were gated as CD45^+^ FSC^low^, SSC^low^, lineage^−^ and ST2/IL-33R^+^ cells (Supplementary Fig. S1a, b). In this study, 87% of ST2/IL-33R^+^ ILC2 were found to be GATA3^+^ and 99% were CD127^+^ (Supplementary Fig. S1a).

At 12 h although no differences in the ST2/IL-33R^+^ ILC2 percentages were detected, significant differences were detected 24 h post vaccination (Fig. 1b). Interestingly, compared to the control unadjuvanted vaccine, the IL-13Rα2-adjuvanted vaccine sequestering IL-13 in the cell milieu, showed significant suppression of ILC2 at the lung mucosae 24 h to 7 days post vaccination, suggesting a requirement for free IL-13 in maintaining ILC2 cells. In contrast, IL-4R antagonist vaccine that blocked both IL-4 and IL-13 cell-signalling via IL-4R/STAT6 pathway showed significantly elevated percentages of ST2/IL-33R^+^ ILC2, 24 h post vaccination compared to the other two vaccines tested (Fig. 1b). It is noteworthy that very low ILC2 were detected in naive mice lung, average 0.074% (Supplementary Fig. S2) vs. unadjuvanted vaccinated 0.29%.

When IL-13 expression was evaluated in the lung lineage^−^ ST2/IL-33R^+^ ILC2, higher IL-13 expression was detected in mice that received the unadjuvanted vaccine compared to the IL-4R antagonist or IL-13Rα2 vaccines (*p* < 0.0001) (Fig. 1c). Interestingly, although IL-4R antagonist and IL-13Rα2 vaccines activated different overall percentages of ST2/IL-33R^+^ ILC2 at the lung mucosae, both vaccines significantly inhibited IL-13 expression by ST2/IL-33R^+^ ILC2, at 24 h to 7 days post vaccination compared to the control unadjuvanted FPV-HIV vaccine (Fig. 1d). It is noteworthy that the trend of ST2/IL-33R^+^ IL-13^+^ cells observed over time presented as percentage or total cell number were similar (Fig. 1d). In all three vaccinated groups, lineage^−^ ST2/IL-33R^+^ ILC2 did not express IL-4 at any of the time points tested. Furthermore, lineage^−^ ST2/IL-33R^+^ ILC2 obtained from unimmunised controls (Supplementary Fig. S2) and importantly the lineage^+^ ST2/IL-33R^+^ cells obtained from vaccinated and non-vaccinated groups (Fig. S3a) also did not show any expression of IL-4 or IL-13. This was further confirmed by staining each lineage marker separately for IL-4 and IL-13 (Supplementary Fig. S3b), and data clearly indicated that the lineage^−^ ST2/IL-33R^+^ cells did not contain any contaminating mast cells or basophils.

Stem cell marker Sca-1 expression^29^ was found to be inversely related to the percentage of CD45^+^ lineage^−^ ST2/IL-33R^+^ ILC2 over time regardless of adjuvant treatment (Fig. 1e, f). Few Sca-1^+^ ILC2 were detected at 12 h in all three vaccine groups tested, however, by 24 h post vaccination CD45^+^ lineage^−^ ST2/IL-33R^+^ ILC2s following IL-4R antagonist-adjuvanted vaccine showed significantly elevated Sca-1 expression compared to the other two vaccines. Although Sca-1^−^ subset did not express IL-13, the Sca-1^+^ ST2/IL-33R^+^ ILC2 subset was positive for IL-13 in the unadjuvanted vaccine, while the IL-4R antagonist and IL-13Rα2-adjuvanted vaccines showed complete inhibition of IL-13 expression (Fig. 1h, g). These results indicated that Sca-1 is a general activation marker for ST2/IL-33R^+^ ILC2, and not necessary dependent on IL-13 expression.

### Expression of IFN-γ and IL-22 by NKp46^+^ ILC subset is differentially regulated following FPV-HIV-IL-13Rα2 and FPV-HIV-IL-4R antagonist vaccination at the lung mucosae

According to the micro environment/cell milieu, high plasticity of ILC1 and ILC3 has been observed and classifying ILC1 and ILC3 according to their cell surface marker expression has been a difficult task.^30–33^ Thus, in this viral vector-based vaccination study, for better clarity the ILC subsets (ILC1 and ILC3) were identified as lineage^−^ NKp46^+^ ILC and lineage^−^ NKp46^−^ ILC, and assessed according to their cytokines production. Unlike the lineage^−^ ST2/IL-33R^+^ cells that were CD127^+^ and GATA3^+^, 99.7% of the lineage^−^ ST2/IL-33R^−^ cells were found to be CD127^−^ and GATA3^−^ (Supplementary Fig. S1a). Furthermore, to confirm that the ILCs were not conventional NK cells, granzyme B expression was evaluated on the lineage^+^ NKp46^+^ and lineage^−^ NKp46^+^ subsets (Supplementary Fig. S4b, d), as expected in the lineage^−^ population, no granzyme B was detected (Supplementary Fig. S4b), whereas in the lineage^+^ NKp46^+^ population, both IFN-γ and granzyme B were detected (Supplementary Fig. S4e). These data clearly confirmed that no conventional NK cells were present in the lineage^−^ population. Interestingly, 24 h post vaccination, the IL-4R antagonist vaccinated group showed elevated IFN-γ expression by the lineage^+^ NKp46^+^ (conventional NK cells) subset (~22%) compared to the control unadjuvanted (~15%) or IL-13Rα2-adjuvanted vaccine groups tested (~7%) (Supplementary Fig. S5a, b). Interestingly, very low NKp46^+^ ILC were detected in naive mice lung, average 0.21% (Supplementary Fig. S2) vs unadjuvanted vaccinated 3.95%.

**Fig. 2:**
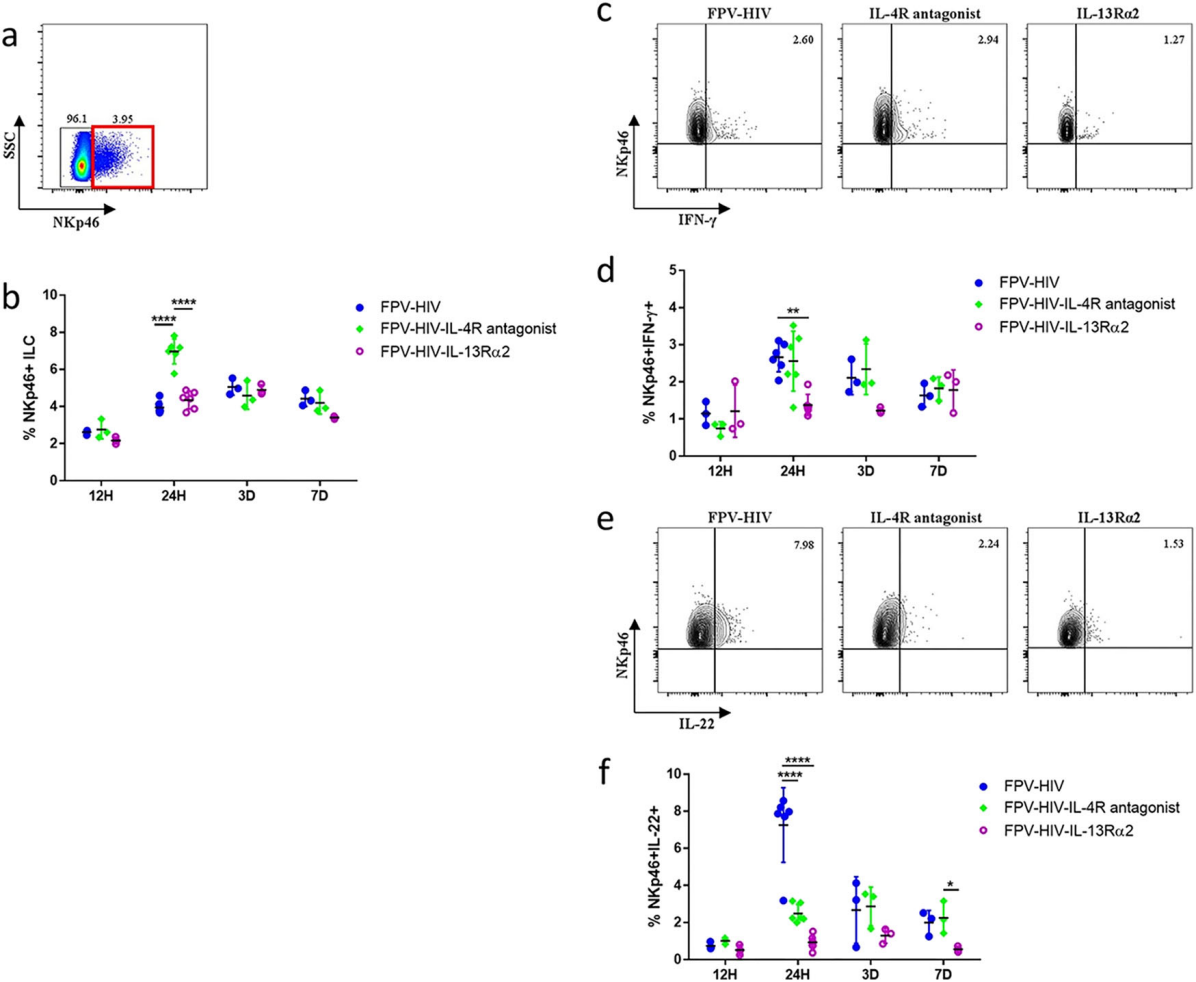
Expression of IFN-γ and IL-22 by NKp46^+^ ILC following i.n. rFPV immunisation. BALB/c mice were immunised intranasally with FPV-HIV, FPV-HIV-IL-4 antagonist, and FPV-HIV-IL-13Rα2 vaccines and the percentage of lung NKp46^+^ ILC cells were evaluated at 12 h, 24 h, 3 days, and 7 days post vaccination. Cells were gated as CD45^+^, FSC^low^, SSC^low^, lineage^−^, ST2/IL-33R^−^ NKp46^+^ (**a**, **b**), and IFN-γ (**c**, **d**) and IL-22 (**e**, **f**) expression were evaluated using intracellular cytokine staining. The graphs represent the mean and standard deviation (s.d.). The *p*-values were calculated using GraphPad Prism software (version 6.05 for Windows). *p < 0.05, **p < 0.01, ****p < 0.0001 (one-way ANOVA). For each time point, experiments were repeated minimum three times

Lineage^−^ ST2/IL-33R^−^ NKp46^+^ ILC were detected in all three vaccine groups tested 24 h post vaccination (Fig. 2b), but the highest percentage was detected in the IL-4R antagonist vaccinated group (*p* < 0.0001) (Fig. 2b). In the context of IFN-γ expression by NKp46^+^ ILC, control unadjuvanted and IL-4R antagonist vaccinated groups showed similar IFN-γ expression profile compared to the lower IL-13Rα2-adjuvanted vaccine group, although only control unadjuvanted showed statistical significance to IL-13Rα2-adjuvanted vaccine (*p* < 0.01) (Fig. 2c, d). Unlike IFN-γ, the IL-22 production by NKp46^+^ ILCs was significantly reduced in both IL-4R antagonist and IL-13Rα2 vaccine groups compared to the control at 24 h (*p* < 0.0001) (Fig. 2e). However, at all time points tested the level of IL-22 was much lower in animals that received FPV-HIV-IL-13Rα2 vaccine (Fig. 2f).

### FPV-HIV-IL-4R antagonist vaccine significantly increases IFN-γ production by NKp46^-^ ILC at the lung mucosae

**Fig. 3:**
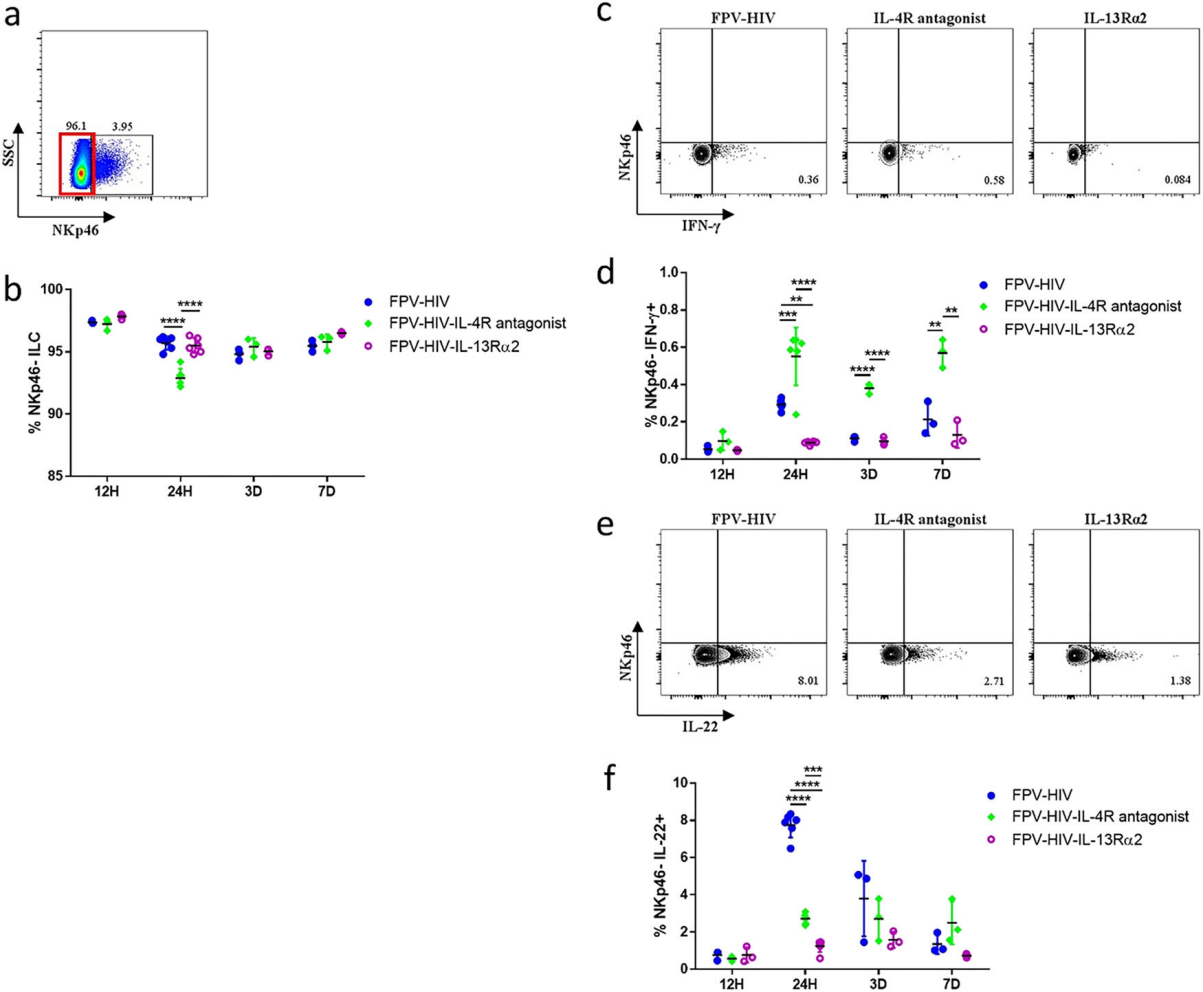
Expression of IFN-γ and IL-22 by NKp46^−^ ILC cells post rFPV immunisation. BALB/c mice were immunised intranasally with FPV-HIV, FPV-HIV-IL-4 antagonist, and FPV-HIV-IL-13Rα2 vaccines and the percentage of lung NKp46^−^ ILC cells were evaluated at 12 h, 24 h, 3 days, and 7 days post vaccination. Cells were gated as CD45^+^, FSC^low^, SSC^low^, lineage^−^, ST2/IL-33R^−^ NKp46^−^ (**a**, **b**), and IFN-γ (**c**, **d**) and IL-22 (**e**, **f**) expression were evaluated using intracellular cytokine staining. The graphs represent the mean and standard deviation (s.d.). The *p*-values were calculated using GraphPad Prism software (version 6.05 for Windows). *p < 0.05, **p < 0.01, ****p < 0.0001 (one-way ANOVA). For each time point experiments were repeated minimum three times

When the lineage^−^ ILCs were assessed post i.n. delivery, most of the cells were found to be ST2/IL-33R^−^ NKp46^−^ ILCs (Fig. 3a). There was no significant difference in the percentages of NKp46^−^ ILC numbers between the vaccine groups tested, except for 24 h time point (Fig. 3b). Next when cytokine expression was evaluated in ST2/IL-33R^−^NKp46^−^ ILCs, significantly elevated IFN-γ expression was detected in FPV-HIV-IL-4R antagonist group compared to the control unadjuvanted group and FPV-HIV-IL-13Rα2 group from 24 h to 7 days post vaccination (*p* < 0.0001–0.01) (Fig. 3c, d). At 24 h post vaccination, similar to the ST2/IL-33R^−^ NKp46^+^ ILC, IL-22 production by ST2/IL-33R^−^ NKp46^−^ ILC was significantly lower in both FPV-HIV-IL-4R antagonist and FPV-HIV-IL-13Rα2-adjuvanted vaccine groups compared to the control unadjuvanted group (Fig. 3e, f).

### Intramuscular vaccination induces exclusive lineage^−^ IL-25R^+^ ILC2 subset at the vaccination site

**Fig. 4:**
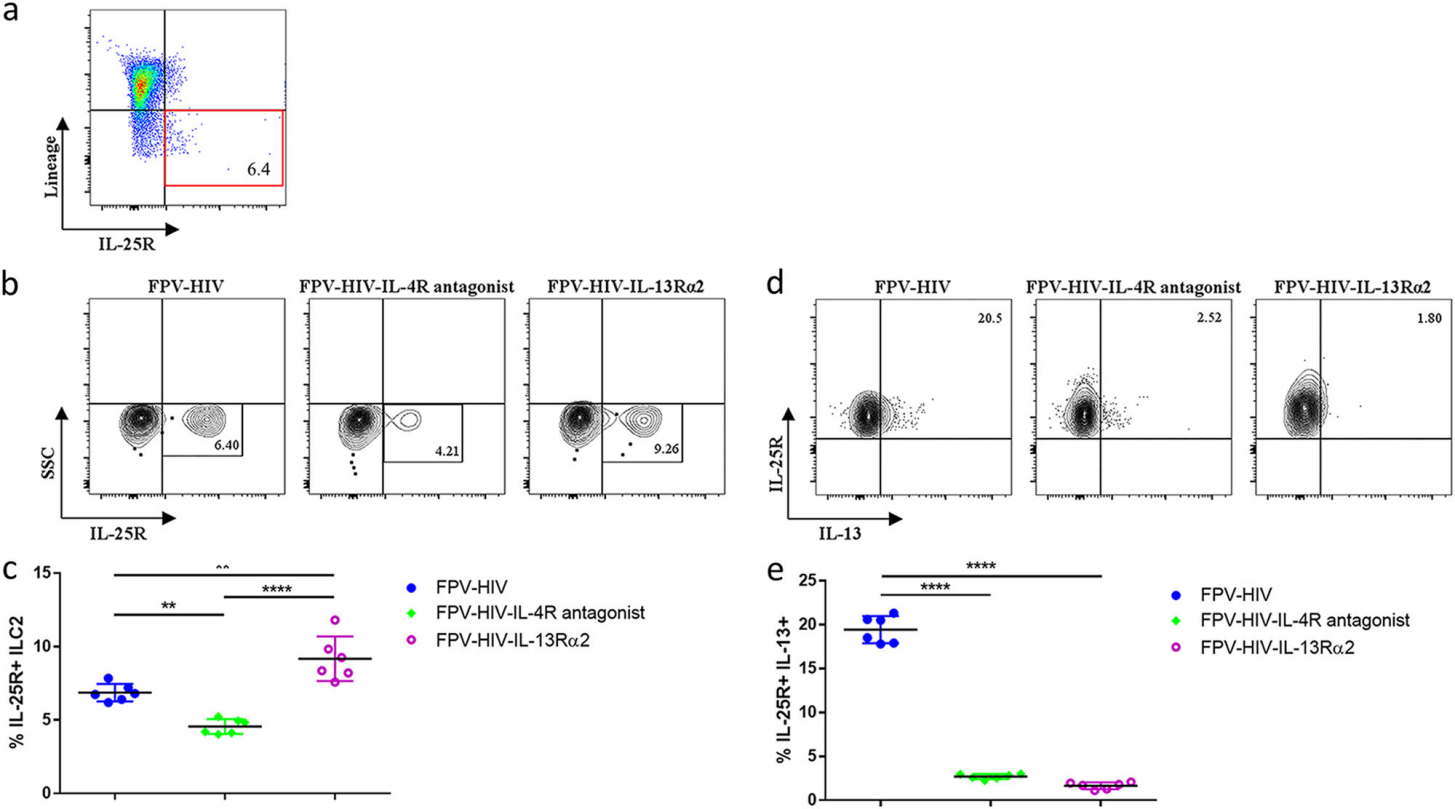
Evaluation of ILC2s and their IL-13 expression in the quadriceps muscle 24 h post intramuscular vaccination. BALB/c mice were immunised intramuscularly with FPV-HIV, FPV-HIV-IL-4 antagonist, and FPV-HIV-IL-13Rα2 vaccines and the percentage of ILC2 in muscle cells were evaluated 24 h post vaccination (note that no ST2/IL-33R^+^ ILC2 were detected in muscle). In this study, ILC2 cells were identified as CD45^+^ FSC^low^ SSC^low^ lineage^−^ ST2/IL-33R^−^ IL-25R^+^ cells using flow cytometry (**a**, **b**). The FACS plots are representative of each vaccination (**b**) and the collected data are presented in graph (**c**). The expression of IL-13 by IL-25R^+^ ILC2s were also assessed (**d**, **e**). The graphs represent mean and standard deviation (s.d.). The *p*-values were calculated using GraphPad Prism software (version 6.05 for Windows). *p < 0.05, **p < 0.01, ****P < 0.0001 (one-way ANOVA). For each time point, experiments were repeated three times. See Supplementary Fig. S1c for gating strategy

Next, when BALB/c mice were immunised intramuscularly (i.m.) and ILC2s were evaluated, no IL-33R^+^ ILC2s were detected in muscle and only IL-25R^+^ ILC2 were observed (Fig. 4a, b). ST2/IL-33R^+^ ILC2s were only found in the lung following intranasal vaccination (Supplementary Fig. S6). However, no IL-25R^+^ or IL-33R^+^ ILC2s were detected in naive quadriceps muscle of BALB/c mice (Supplementary Fig. S7). More interestingly, compared to the other two vaccines, FPV-HIV-IL-4R antagonist vaccine significantly supressed the IL-25R^+^ ILC2s while FPV-HIV-IL-13Rα2-adjuvanted vaccine significantly increased the IL-25R^+^ ILC2 number (Fig. 4c). When IL-13 expression by lineage^−^ ST2/IL-33R^−^ IL-25R^+^ ILC2 subset was assessed, significantly higher IL-13 expression was detected in control unadjuvanted vaccines compared to the FPV-HIV-IL-4R antagonist and FPV-HIV-IL-13Rα2-adjuvanted vaccines (*p* < 0.0001) (Fig. 4d). Interestingly, although FPV-HIV-IL-4R antagonist and FPV-HIV-IL-13Rα2-adjuvanted vaccines activated different overall percentages of IL-25R^+^ ILC2 numbers, both vaccines were able to significantly downregulate the IL-13 production by IL-25R^+^ ILC2 compared to control unadjuvanted FPV-HIV vaccine (Fig. 4e). In all vaccine groups tested, no IL-4 production was detected in IL-25R^+^ ILC2.

### Intramuscular vaccination induces uniquely different lineage^−^ NKp46^-^ and NKp46^+^ ILC subsets at the vaccination site

**Fig. 5:**
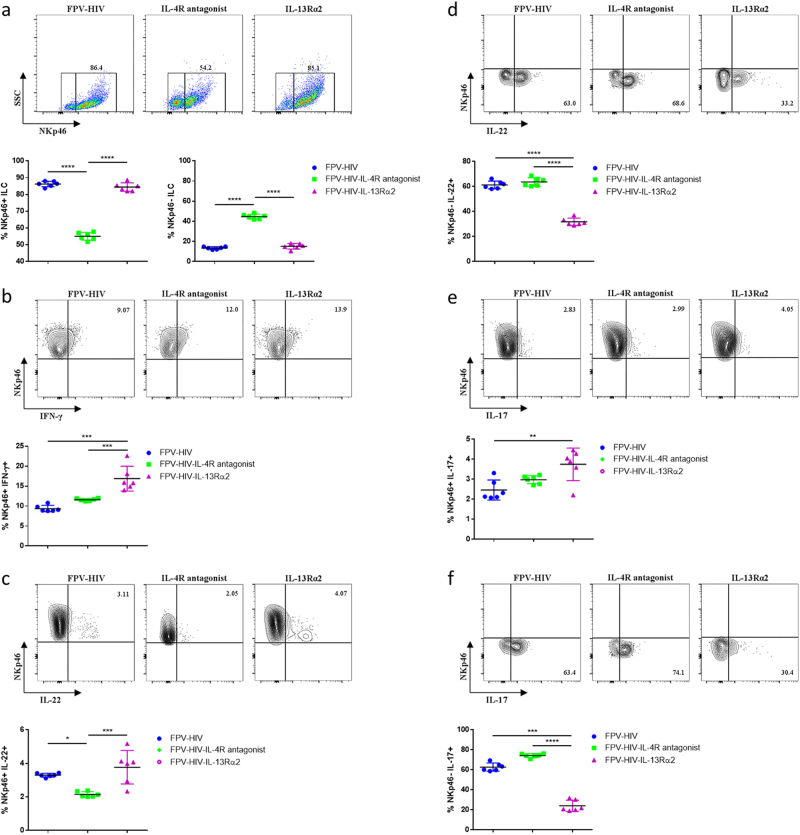
Evaluation of NKp46^+/-^ ILC cytokines expression in the quadriceps muscle 24 h post intramuscular vaccination. BALB/c mice were immunised intramuscularly with FPV-HIV, FPV-HIV-IL-4 antagonist, and FPV-HIV-IL-13Rα2 vaccines, and the percentage of NKp46^+^ and NKp46^−^ ILC in muscle cells were evaluated 24 h post vaccination. The NKp46^+^ and NKp46^−^ ILCs were evaluated exactly as per lung tissue (**a**). Data represent percentage NKp46^−^and/or NKp46^+^ cells expressing IFN-γ (**b**), IL-22 (**c**, **d**) and IL-17A (**e**, **f**). The graphs represent the mean and standard deviation (s.d.). The *p*-values were calculated using GraphPad Prism software (version 6.05 for Windows). *p < 0.05, **p < 0.01, ****p < 0.0001 (one-way ANOVA). For each time point experiments were repeated three times

When lineage^−^ IL-25R^−^ (also ST2/IL-33R^−^) NKp46^+^ and NKp46^−^ ILC subsets were evaluated 24 h post vaccination, the FPV-HIV-IL-4R antagonist vaccinated group showed very low NKp46^+^ ILC and elevated percentage of NKp46^−^ ILC compared to the control unadjuvanted and FPV-HIV-IL-13Rα2 vaccines (Fig. 5a). Interestingly, in naive mice very low NKp46^+^ ILC were detected compared to unadjuvanted vaccinated, average 2.65% (Supplementary Fig. S7) vs. 86.4%. When cytokine profiles were evaluated in these two subsets, significantly elevated IFN-γ and IL-22 expression was detected in NKp46^+^ ILC obtained from FPV-HIV-IL-13Rα2-adjuvanted vaccinated group compared to the IL-4R antagonist vaccine group (*p* < 0.05–0.0001) (Fig. 5b, c). Also, the percentage of IL-25R^−^ NKp46^+^ ILC that expressed IFN-γ (*p* < 0.001) and IL-17A (*p* < 0.01) was significantly higher in FPV-HIV-IL-13Rα2-adjuvanted group compared to the control (Fig. 5b–e). Overall, the HIV-IL-13Rα2 vaccinated group showed elevated percentage of lineage^−^ IL-25R^−^ NKp46^+^ ILC-expressing IFN-γ, IL-22 and IL-17A compared to the other two groups tested.

Unlike IL-22 and IL-17A that was detected in both lineage^−^ IL-25R^−^ NKp46^+^ and NKp46^−^ ILC subsets, no IFN-γ expression was detected IL-25R^−^ NKp46^−^ ILC. Although there was no significant difference in IL-22 and IL-17A production by NKp46^−^ ILC subsets between control unadjuvanted and FPV-HIV-IL-4R antagonist vaccinated groups, FPV-HIV-IL-13Rα2-adjuvanted vaccinated group showed significantly reduced percentage of NKp46^−^ ILC-expressing IL-17A compared to the other two vaccine groups tested (*p* < 0.001–0.0001) (Fig. 5d, f). No NKp46^+^ or NKp46^−^ ILC subsets were positive for both IL-22 and IL-17A.

## Discussion

In this study, our approach of characterising lineage^−^ ILC subsets based principally upon cytokine expression without preconceived expectations of cell surface markers have shown that ILC induced following viral vector vaccination are uniquely different to what has been reported under experimentally induced or chronic inflammation conditions. Following rFPV vaccination although ILC2 were found to express CD127, NKp46^+^ and NKp46^−^ ILC1, and ILC3 populations did not express CD127. This was not entirely surprising as a recent study had shown that CD127 was not strictly required for the development of any ILC subsets.^34^ These observations highlight the caveats of using conventional flow cytometry analysis that relies only on surface marker expression (i.e., Sca-1, CD127 or NKp46) to study ILCs under different conditions (i.e., vaccination or acute infection), especially given the high plasticity of ILC subsets.^9–11,30–33^

Different ILC2 subsets arise from a common progenitor cell and under different cytokine conditions/anatomical location differentiate into ILC2 that are ST2/IL-33R^+^, IL-25R^+^ or TSLPR^+^.^35^ Here, we have for the first time demonstrated that route of vaccination can alter the ILC recruitment profile at the vaccination site. Following i.n. rFPV vaccination only ST2/IL-33R^+^ ILC2 (lung resident “natural” ILC) were detected in lung, while exclusively IL-25R^+^ ILC2s were detected in muscle following i.m. rFPV vaccination. IL-25R^+^ ILC2 in muscle were most likely circulatory “inflammatory” ILC2^36^ as naive muscle did not show any significant ILC2 subsets (Supplementary Fig. S7). Both lung ST2/IL-33R^+^ ILC2s and muscle IL-25R^+^ ILC2s although expressed IL-13, did not express IL-4. As lineage^+^ cells did not express any IL-13 at 12 h or 24 h post vaccination, ILC2 were the only or major source of IL-13 at the vaccination site at these specific time points. Interestingly, a larger proportion of IL-13^+^ ILC2s were detected in muscle following i.m. FPV-HIV vaccination compared to lung post i.n. delivery. These observations revealed that what is observed at the ILC level could be directly translated into T-cell level, where intramuscular poxviral vaccination has shown to induce elevated IL-13 expression by CTL responsible for low avidity T cells compared to mucosal delivery.^37^

Moreover, IL-13Rα2 and IL-4R antagonist-adjuvanted vaccines that induced high-avidity CTL^19,20^ showed significant inhibition of IL-13 expression by lung ST2/IL-33R^+^ ILC2 compared to control non-adjuvant vaccination. Taken together, the ability of IL-13 to modulate dendritic cell recruitment to the vaccination site 24 h post vaccination,^18^ current findings clearly propose that level of IL-13 expressed by ILC2, could directly modulate dendritic cell recruitment to the vaccination site, responsible for the generation of uniquely different T-cell immune outcomes (i.e., under low IL-13 condition recruitment of CD11b^+^ CD103^−^ conventional DC responsible for induction high-avidity CTLs^18^). Interestingly, under allergic lung inflammation conditions IL-13 but not IL-4 expressed by ILC2s have shown to promote migration of activated lung dendritic cells into the draining lymph nodes responsible for CD4^+^ T-helper 2 cell activation.^38^ Halim et al.^17^ have further demonstrated that IL-13^+^ ILC2 licence CD11b^+^ CD103^−^ conventional DC to express CCL17 and promotes Th2 responses, and our studies showed that blocking IL-13 activity likely inhibit this process and forces stronger Th1-mediated immunity.^28^ In summary, these findings indicate that the ILC2 bias (IL-25R^+^ or IL-33R^+^) observed at the different vaccination sites and the amount of IL-13 produced by these ILC2, play a critical role in defining the efficacy of a vaccine. This may also explain how and why mucosal vaccination (which induce low IL-13 expression by ILC2) induce high-avidity CTL with better protective efficacy against mucosal pathogens compared to systemic vaccination.^37,39,40^

IL-13 and IL-4 share a common receptor system comprised of IL-4Rα/IL-13Rα1, Type II IL-4 receptor complex,^41,42^ while IL-4 can also signal via Type I IL-4 receptor complex comprised of the common-γ chain and IL-4Rα.^43^ Both these receptor complexes activate STAT6 signalling. IL-13Rα2 and IL-4R antagonist-adjuvanted vaccines inhibited IL-13 expression by ILC2s compared to the control non-adjuvanted vaccine. Sequestering IL-13 (IL-13Rα2-adjuvanted vaccination) also reduced the overall percentage of ST2/IL-33R^+^ ILC2 suggesting an IL-13 autocrine role in maintaining ILC2 function. Surprisingly, sequestering IL-13 from the milieu vs. blocking conventional IL-4Rα/IL-13Rα1/STAT6 signalling using IL-4R antagonist vaccines resulted in differing ILC1/ILC2 responses. These observations indicate that IL-13 signalling via an alternative pathway, most likely IL-13Rα2 may possibly be responsible for the responses observed with the latter vaccination strategy. This can be further corroborated by the findings that under certain conditions, a physical interaction between cytoplasmic domains of IL-13Rα2 and IL-4Rα regulating IL-4Rα/IL-13Rα1 receptor function,^44^ and also IL-13 signalling via the not well defined high-affinity IL-13Rα2 pathway has been reported.^45^

Furthermore, IL-13Rα2-adjuvanted vaccination was associated with lower IFN-γ expression by both NKp46^+/-^ ILC and conventional NK cell (Supplementary Fig. S5) compared to the other two vaccines tested. Interestingly, a recent study has demonstrated that IL-1β, IL-12 and IL-18 drive the plasticity between ILC2 and IFN-γ^+^ ILC1 populations.^46^ Thus, the very low starting population of ILC2 induced under IL-13Rα2-adjuvanted vaccine may account for the reduced IFN-γ^+^ NKp46^+/-^ ILC populations. However, whether this would directly affect the IFN-γ expression by conventional NK cell is not yet known (Supplementary Fig. S5). In contrast, our study demonstrated that IFN-γ expression by lung NKp46^−^ ILC was elevated up to the day 7 experimental period, while IFN-γ expression by “ex-ILC3” NKp46^+^ cells was largely unaffected following IL-4R antagonist adjuvant vaccination. It is accepted that IL-4/IL-13/STAT6 signalling is antagonistic to IFN-γ expression.^47^ Thus, it is not entirely surprising that blocking the conventional IL-4Rα/IL-13Rα1 pathway resulted in elevated IFN-γ expression by both NKp46^−^ILC and conventional NK cells. We have previously shown that vaccination using the IL-4R antagonist vaccine resulted in robust IgG1 and IgG2a antibody responses, whereas the IL-13Rα2-adjuvanted vaccine resulted in reduced IgG2a antibodies.^20^ Interestingly, one major difference between the two adjuvanted vaccines were the levels of IFN-γ expression by NKp46^−^ ILC and NK cells suggesting that this may ultimately influence APC activation and CD4^+^ T-helper cells required for antibody isotype class switching. Blocking autocrine signalling via the IL-4R/IL-13Rα1 (blocking STAT6 signalling) may account for reduced ILC2 IL-13^+^ cell number, it has also been shown that ILC1 IFN-γ expression can suppress IL-13 expression by tissue-resident natural ILC2 cells.^48^ IFN-γ has shown to significantly inhibit IL-13 production by ILC2,^48–50^ while also upregulating extracellular expression of IL-13Rα2.^47^ The above and our current findings indicate a complex interaction between IFN-γ and IL-13 signalling at the vaccination site, and the early IL-13 and IFN-γ co-dependency at the ILC level most likely playing an important role in shaping the downstream antibody immunity.

Previous inflammation and asthma mouse models indicate NKp46^−^ ILC1 express IFN-γ, NKp46^−^ ILC3 express IL-17A and NKp46^+^ ILC3 express IL-22.^51–53^ Interestingly, in this study, following intranasal viral vaccination both NKp46^+^ and NKp46^−^ ILC1 and ILC3 were found to express IFN-γ and IL-22, but not IL-17A. In contrast, following intramuscular vaccination NKp46^+^ ILC1 and ILC3 were found to express IFN-γ, IL-22 and IL-17A, while NKp46^−^ ILCs only expressed IL-22 and IL-17A, not IFN-γ. These findings clearly indicate the ILC1/ILC3 and ILC2 populations induced are uniquely different depending upon the route of vaccination. Furthermore, the adjuvanted vaccine studies demonstrated that unlike i.n. delivery, the NKp46^+^ and NKp46^−^ ILC1/ILC3 subsets induced following i.m. vaccination have significantly different responsiveness to IL-13. For example, unadjuvanted and the IL-4R adjuvant vaccines showed significantly elevated IL-22 and IL-17A expression by NKp46^−^ ILC1/ILC3 compared to IL-13Rα2-adjuvanted vaccine. Also, a larger proportion of IL-13^+^ ILC2s were detected following i.m. FPV-HIV vaccination compared to i.n. delivery. Taken together, our previous studies on CD8^+^ T cells, where IL-13 has shown to modulate IL-17A activity,^27^ we postulate that IL-17A expression in ILC1 and ILC3 is tightly regulated by IL-13-driven ILC2. In summary, these observations evoke the possibility that the NKp46^+^ or NKp46^−^ ILC1 and ILC3 plasticity at vaccination site is co-dependent on the amount of IL-13 produced by different ILC2 subsets (IL-25R^+^ vs. IL-33R^+^).

Collectively, results indicate that within the first 24 h post vaccination, according to the route of delivery and adjuvants used, different types of IL-13-driven ILC2 and IFN-γ /IL-17A/IL-22-expressing NKp46^+^ or NKp46^−^(ILC1 and ILC3) are recruited to the vaccination site. The IL-13 and IFN-γ/IL-17A balance induced by these ILCs, play a crucial role in shaping the resulting APC recruitment/activation and B- and T-cell immunity. Our data suggest that altering the functions of these different ILC subsets at the vaccination site, by regulating IL-13 signalling to induce the desired protective immune outcome needed according to the target pathogen (bacteria, viruses or parasites), may give rise to more efficacious vaccines in the future.

## Methods

### Mice

Five to seven-week-old pathogen-free female wild-type (WT) BALB/c mice were obtained from the Australian Phenomics Facility, the Australian National University. All animals were maintained and experiments were performed in accordance with the Australian NHMRC guidelines within the Australian Code of Practice for the Care and Use of Animals for Scientific Purposes and in accordance with guidelines approved by the Australian National University Animal Experimentation and Ethics Committee (AEEC). This study was approved by the AEEC and listed under ANU ethics protocol numbers A2014/14 and A2017/15.

### Immunisation

In this study, 1 × 10^7^ PFU unadjuvanted FPV-HIV vaccine and/or FPV-HIV-IL-4R antagonist vaccine and FPV-HIV-IL-13Rα2-adjuvanted vaccines were given to BALB/c mice i.n. or i.m. The i.n. vaccines were given in 10–15 µl per nostril (total 25–30 µl volume) and i.m. vaccines 50 µl per quadriceps muscle as per described previously.^19,20^ All vaccines were diluted in sterile Phosphate buffered saline (PBS) and sonicated three times 15 s at 50 output using a Branson Sonifier 450 prior to use.

### Preparation of lung and muscle lymphocytes

Lung tissues were collected 12 h, 24 h, 3 days and 7 days post rFPV immunisation. Quadriceps muscles were collected only at 24 h post immunisation. Lung tissues were first cut into small pieces, and then enzymatically digested for 45 min at 37 °C in digestion buffer containing 1 mg/ml collagenase (Sigma-Aldrich, St. Louis, MO), 1.2 mg/ml Dispase (Gibco, Auckland, NZ), 5 Units/ml DNase (Calbiochem, La Jolla, CA) in complete RPMI. Samples were mashed and passed through a falcon cell strainer and resulting lung cell suspensions were then lysed with RBCs, washed and passed through gauze to remove debris as per described previously.^19,20,54^ Muscles were also cut into small pieces and digested with 0.5 mg/ml collagenase, 2.4 mg/ml Dispase, 5 Units/ml DNase and complete RPMI for 30 min at 37 °C, passed through a falcon cell strainer (without mashing to avoid creating smaller debris) and gauze to remove debris similar to lung. The cells were suspended in complete RPMI, rested overnight at 37 °C with 5% CO_2_ as per our previous studies.^19^ No in-vitro stimulation was performed as the purpose was this study was to evaluate in-vivo stimulation state of the viral-induced cytokine production. All cells were treated with 1% Brefeldin A for 5 h and stained and analysed using multi-colour flow cytometry.

### Flow cytometry

Monoclonal antibodies Fluorescein isothiocyanate (FITC)-conjugated anti-mouse CD3 (T cells) clone 17A2, CD19 (B cells) clone 6D5, CD11b (macrophages and dendritic cells) clone M1/70, CD11c (dendritic cells) clone N418, CD49b (NK, NKT, T cells) clone HMα2, FcεRIα (mast cells and basophils) clone MAR-1 (all linage positive markers were selected as FITC), PE-conjugated anti-mouse ST2/IL-33R (clone DIH9), APC-conjugated anti-mouse Sca-1 (clone D7), APC/Cy7-conjugated anti-mouse CD45 (clone 30-F11), Brilliant Violet 605-conjugated anti-mouse CD127 (IL-7R) (clone A7R34), Brilliant Violet 421-conjugated anti-mouse CD335 (NKp46) (clone 29A1.4), APC-conjugated anti-mouse IL-17RB (IL-25R) (clone 9B10), PerCP/Cy5.5-conjugated anti-mouse GATA3 (clone 16E10A23), Brilliant Violet 421-conjugated anti-mouse IL-4 (clone 11B11), Brilliant Violet 510-conjugated anti-mouse IFN-γ (clone XMG1.2), APC-conjugated IL-22 (clone Poly5164), Alexa Fluor 700-conjugated IL-17A (clone TC11-18H10.1) were obtained from BioLegend. PE-eFlour 610-conjugated anti-mouse IL-13 (clone eBio13A) and PE-conjugated anti-mouse Granzyme B (clone 16G6) were purchased from eBioscience. ILC2s and ILC1/3s were stained separately to avoid fluorochrome overlap. Specifically, FITC-conjugated lineage cocktail antibodies and APC/Cy7-conjugated anti-mouse CD45 were used in both ILC2 and ILC1/ILC3 staining. PE-conjugated anti-mouse ST2/IL-33R, APC-conjugated anti-mouse Sca-1, Brilliant Violet 605-conjugated anti-mouse CD127 (IL-7R) (clone A7R34), Brilliant Violet 421-conjugated anti-mouse IL-4, and PE-eFlour 610-conjugated anti-mouse IL-13 were only used in ILC2s staining. Brilliant Violet 421-conjugated anti-mouse NKp46, Brilliant Violet 605-conjugated anti-mouse CD127 (IL-7R) (clone A7R34), Brilliant Violet 510-conjugated anti-mouse IFN-γ, APC-conjugated IL-22, Alexa Fluor 700-conjugated IL-17A were only used in ILC1/3 s staining. The cell surface and intracellular staining were performed according to protocols established in our laboratory,^19^ fixed with 0.5% paraformaldehyde, and run on a BD LSR Fortessa. From each sample 2,00,000 (muscle) or 14,00,000 (lung) events were acquired and data were analysed with Tree Star FlowJo software (version 10.0.7 for Windows). The gating strategy used to identify each ILC subsets are indicated in Supplementary Figures section (Supplementary Fig. S1a–c).

### Statistical analysis

In this study, cell numbers were calculated using the formula (cytokine expressing cells/number of CD45^+^ cells) × 10^6^. The graphs represent the mean and standard deviation (s.d.). The *p*-values were calculated using GraphPad Prism software (version 6.05 for Windows). One-way ANOVA or two-way ANOVA using Tukey’s multiple comparisons test were used to calculate statistical significance. The *p*-values were denoted as follows: ns—*p* ≥ 0.05, *p < 0.05, **p < 0.01, ***p < 0.001, ****p < 0.0001. In this study, *n* = 3 to 6 mice per group were used and all experiments were repeated at least three times, and 24 h experiments were repeated six times.

### Data availability statement

The authors declare that all data supporting the findings of this study are available within the paper and supplementary files.

## Electronic supplementary material

Supplementary figures
