# Supplementary material for: Vaccination route can significantly alter the innate lymphoid cell subsets: a feedback between IL-13 and IFN-γ
Source: NPJ Vaccines. 2018 Mar 12;3:10. doi: 10.1038/s41541-018-0048-6 (PMC5847557; doi:10.1038/s41541-018-0048-6)
Supplement: Supplementary file 1 — Supplementary figures [file 41541_2018_48_MOESM1_ESM.docx]

**Supplementary Figures**:

**Fig. S1a. Lung ILC gating strategy**


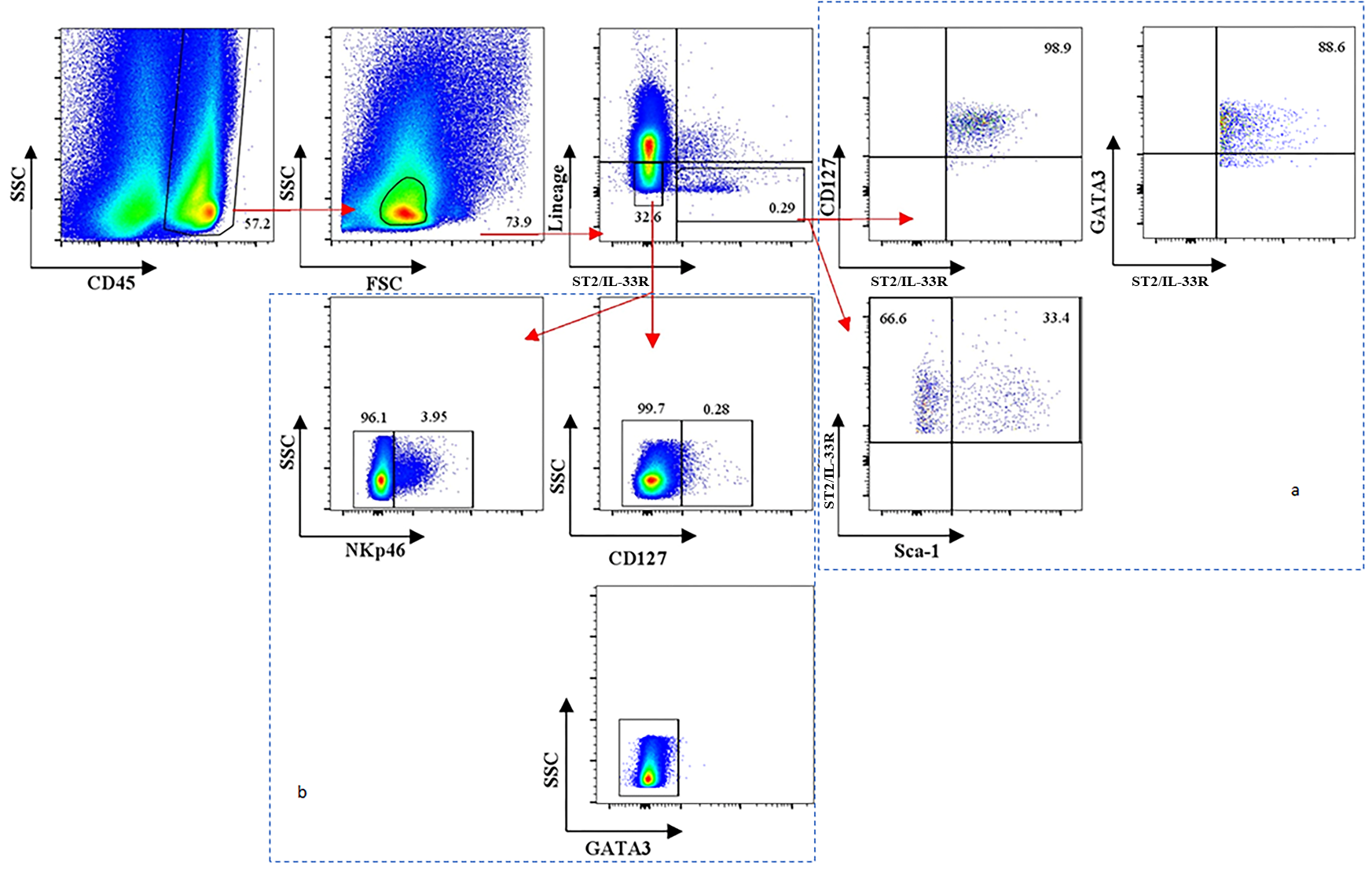


Lung ILCs from BALB/c mice were evaluated 24h post FPV-HIV immunization as per described in Materials and Methods. Firstly, from total lung cells, CD45^+^ cells and lymphocytes were gated. Next within the lymphocyte gate, ILC2 were identified as lineage^-^ ST2/IL-33R^+^ cells. When Sca-1, CD127 (IL-7R) and GATA3 expression profiles were evaluated on lineage^-^ cells, while most ST2/IL-33R^+^ cells were CD127^+^ (99%) and GATA3^+^ (88.6%), only 33% cells were found to be Sca-1^+^ **(a)**. When the lineage^-^ ST2/IL-33R^-^ cells (ILC1 and ILC3) were stained with NKp46, 96% cells were NKp46^-^ while only 4% were NKp46^+^ **(b)**. Interestingly, following vaccination most of the ILC1 and ILC3 cells were found to be CD127^-^ (99%) and all of the lineage^-^ ST2/IL-33R^-^ cells were GATA3^-^ **(b)**.

These data clearly revealed that although under chronic inflammatory condition, CD127 is considered as a ubiquitous marker for fully differentiated ILCs, following acute infection or vaccination the expression of CD127 on ILC can be fluid and dependent upon the level of ILC differentiation.

**Fig. S1b. Single colour controls and FMOs for lineage cells and ST2/IL-33R^+^ cells**


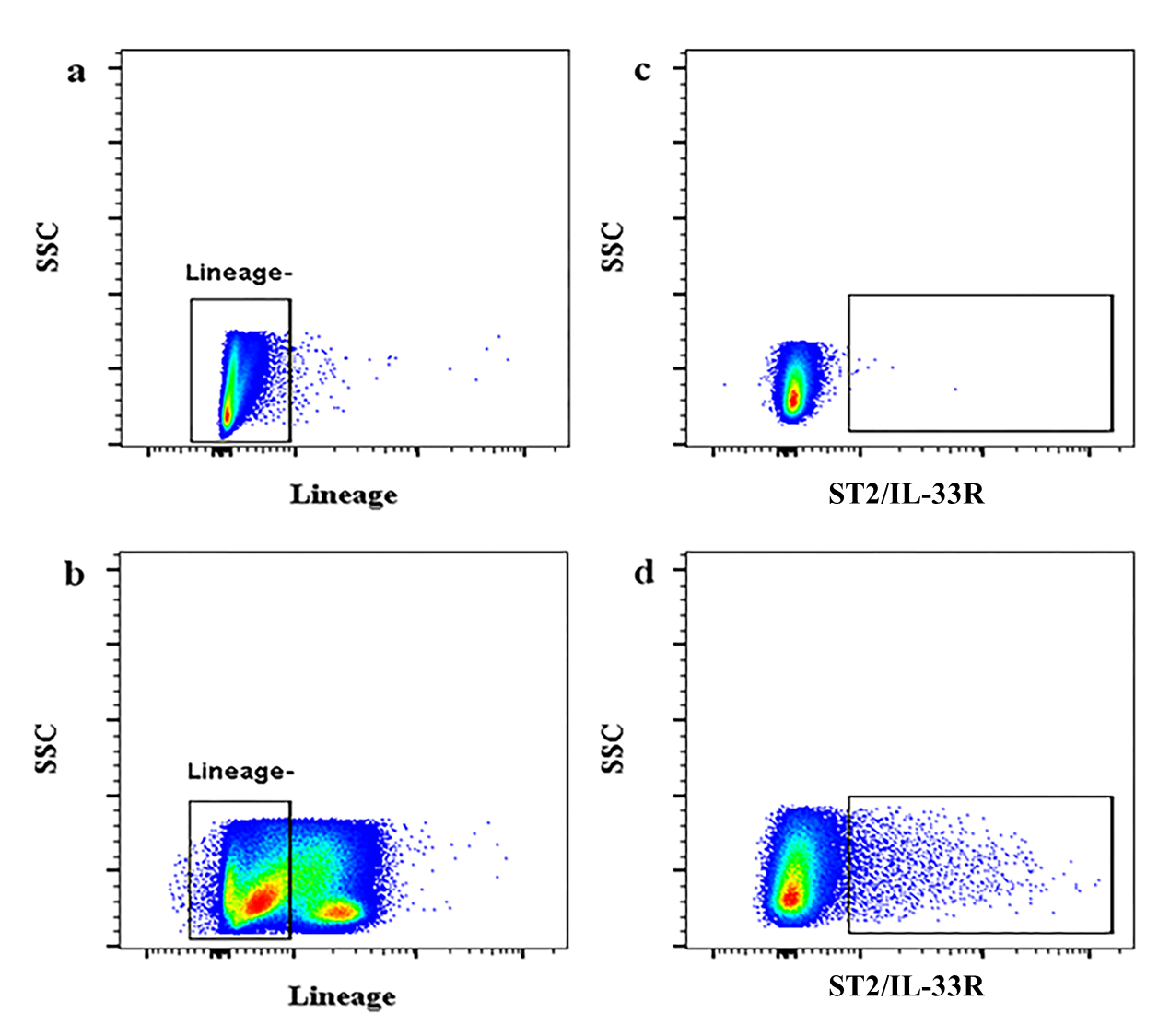


The gating strategy for lineage^-^ and ST2/IL-33R^+^ population was based on FMO and single colour controls. FMO for lineage cells **(a)**, Lineage+ single colour control **(b)**, FMO for ST2/IL-33R cells **(c)** and ST2/IL-33R single colour control **(d).**

**Fig. S1c. Quadriceps muscle ILC gating strategy**


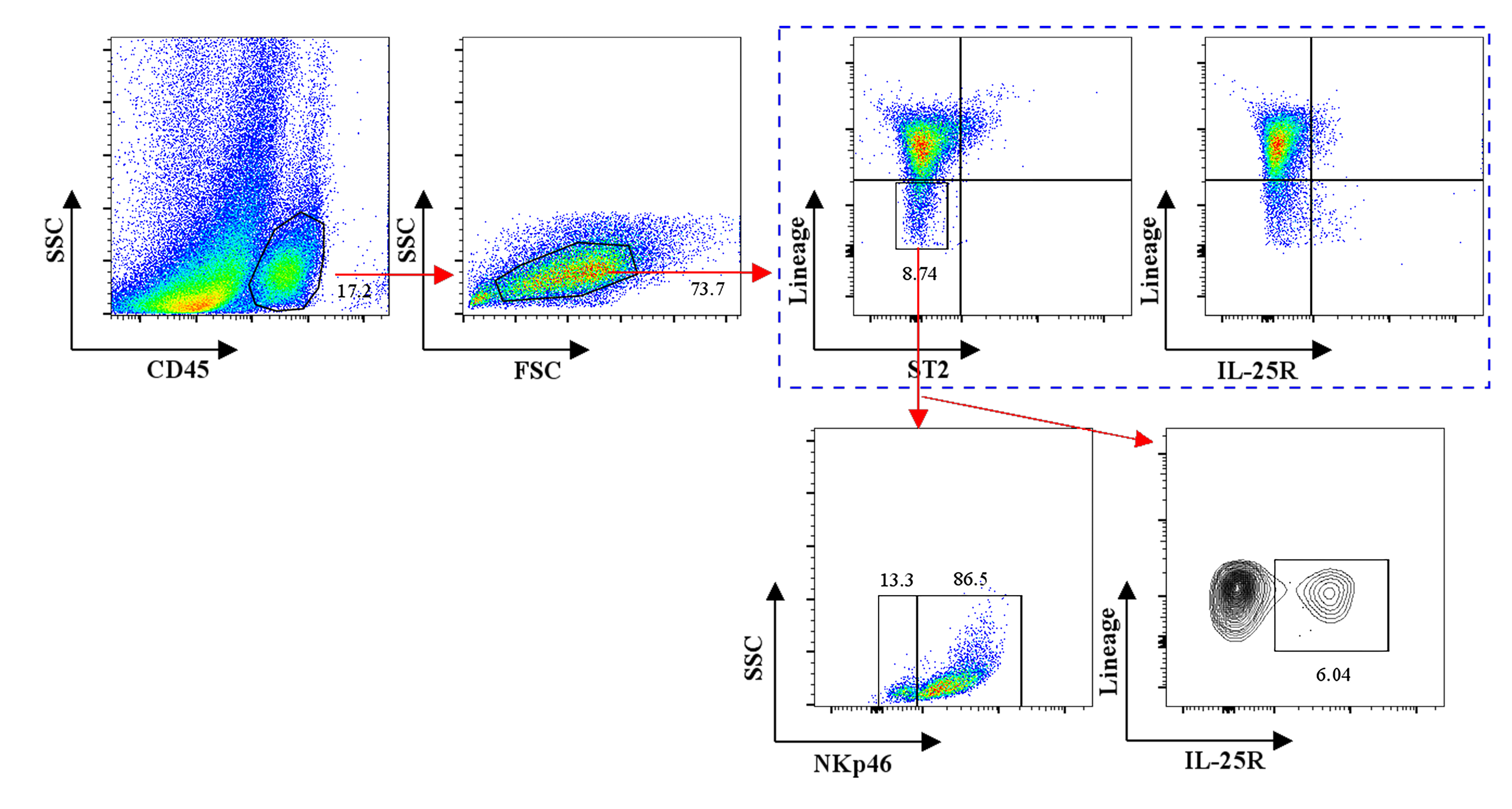


Muscle ILCs from BALB/c mice were evaluated 24h post FPV-HIV immunization. From total muscle cells, CD45^+^ cells and lymphocytes were gated as per lung, and assessed for lineage^-^ ST2/IL-33R^+^ and lineage^-^ IL-25R^+^ cells. Data revealed that lineage^-^ ST2/IL-33R^-^ cells (ILC1& ILC3), 86% cells were NKp46^+^ while only 13% were NKp46^-^. Note that the lineage- IL-25R+ cell number in both top and bottom right FACS plots were the same.

**Fig. S2. Evaluation of IL-13, IFN-γ, and IL-22 expression by different ILC subsets in naïve BALB/c mice**


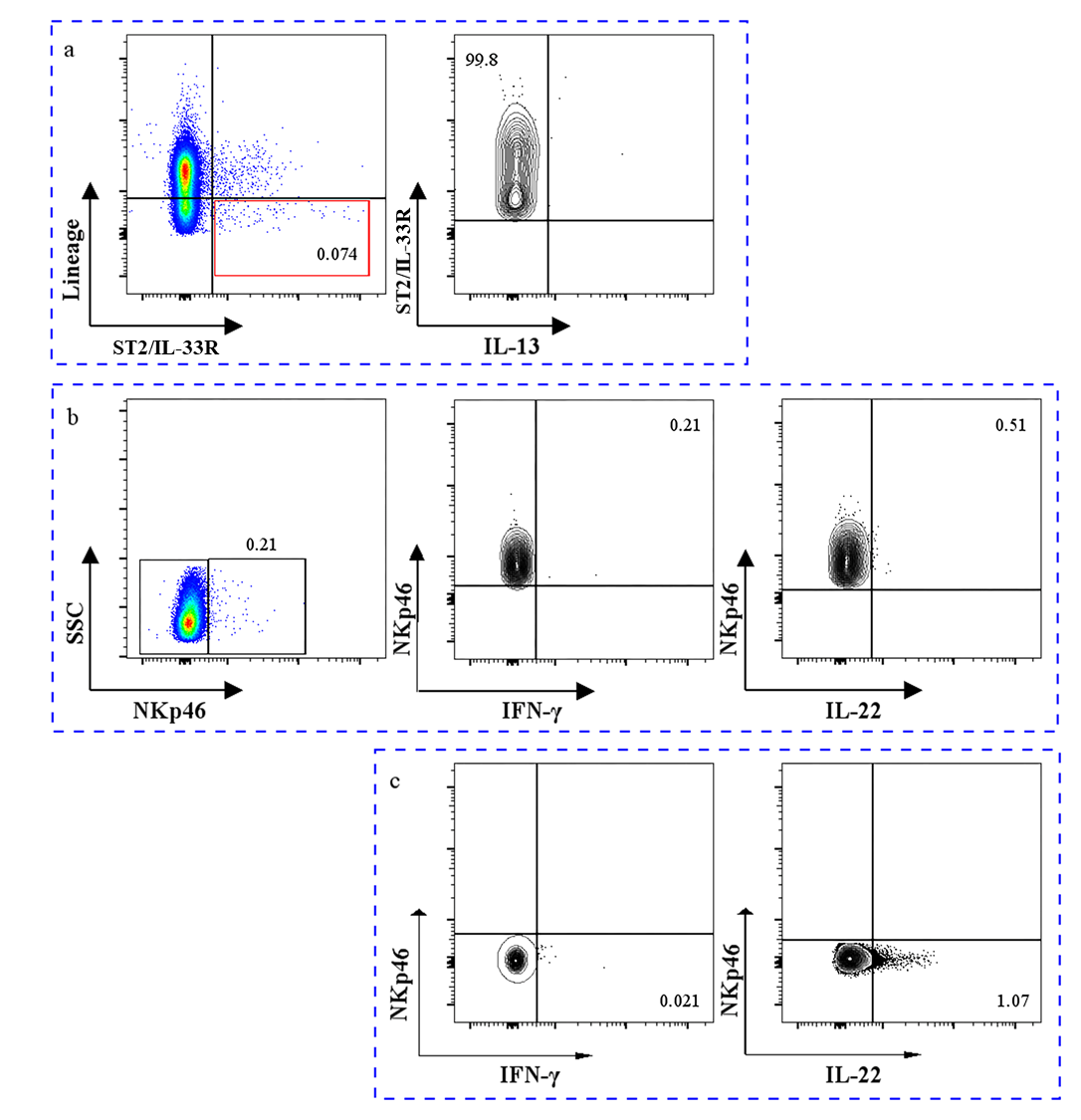


Lung ILC from naïve BALB/c mice were prepared and stained as described in Materials and Methods. In naïve mice no IL-13 expression by ILC2 cells were detected **(a)**. Although no IFN-γ expression was detected in NKp46^+^ and NKp46^-^ ILC1 and ILC3 **(b)** some IL-22 expression was detected in NKp46^-^ ILC1 and ILC3 **(c)**.

**Fig. S3a. Evaluation of IL-13 expression by lung lineage^+^ ST2/IL-33R^+^ cells following i.n. rFPV immunization**

**
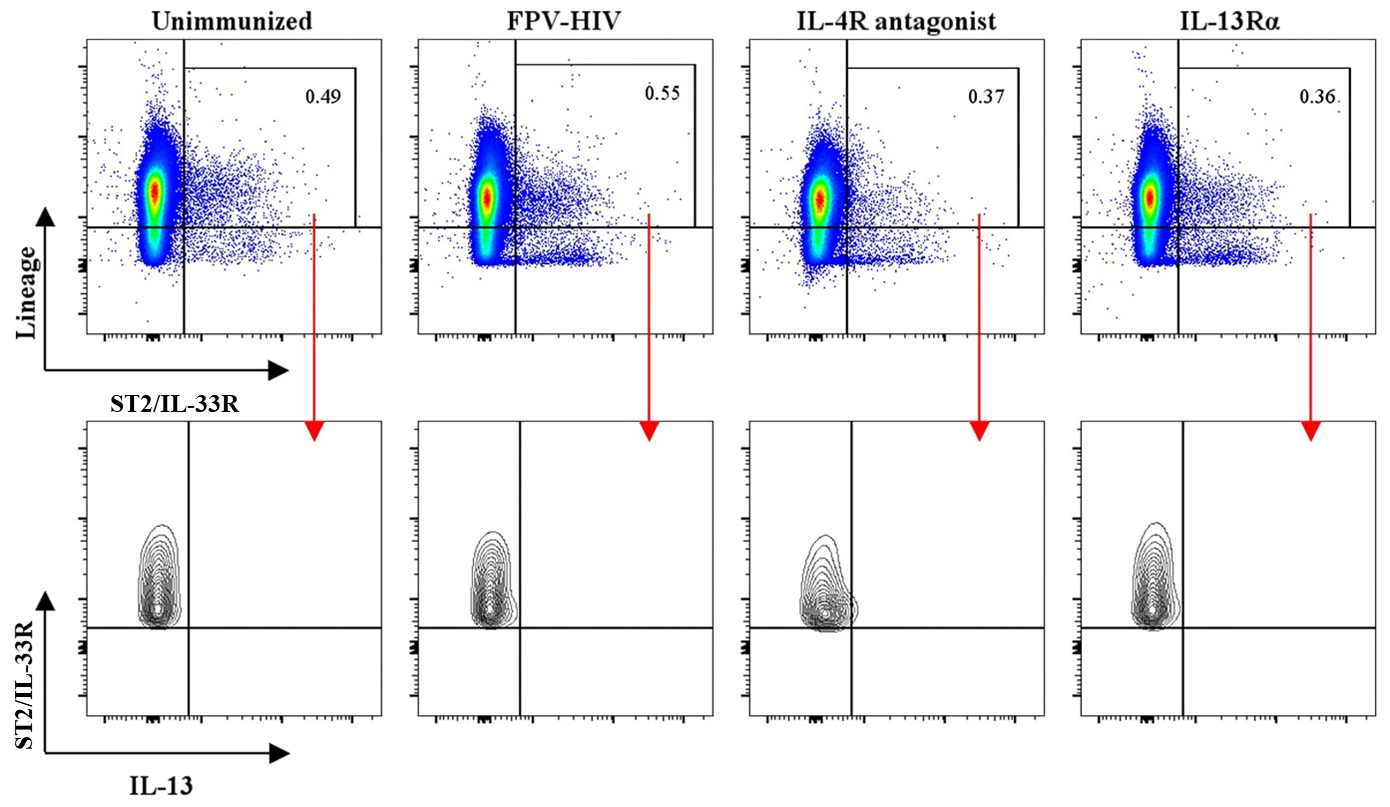
**

BALB/c mice were immunized intranasally with FPV-HIV, FPV-HIV-IL-4R antagonist adjuvanted, and FPV-HIV-IL-13Rα2 adjuvanted vaccines and IL-13 expression by lineage^+^ ST2/IL-33R^+^ cells were evaluated at 24h post vaccination and compared with unimmunized naïve mice. Data indicated that no IL-13 expression (0%) was detected in lineage^+^ ST2/IL-33R^+^ cells under all three vaccine conditions.

**Fig. S3b. Evaluation of IL-13 and IL-4 expression by each lineage^+^ cell subset following i.n. rPFV immunization**


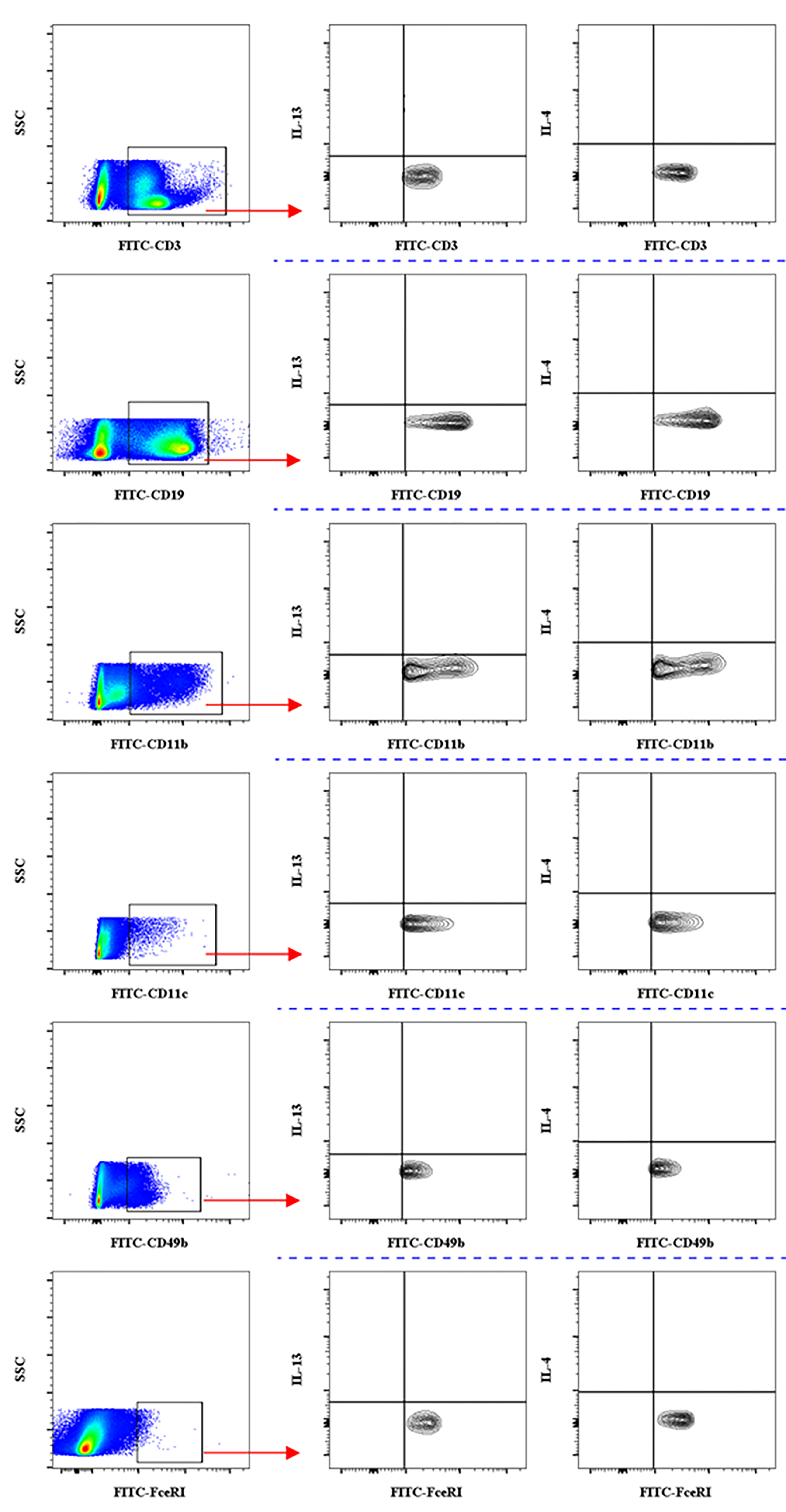


BALB/c mice were immunized intranasally with FPV-HIV vaccine, lung cells were stained with each lineage marker separately. IL-13, and IL-4 expression were evaluated at 24h post vaccination. Data revealed that none of the lineage^+^ cell subsets expressed IL-13 or IL-4, further confirming that the ILC2 cells were not contaminated with any lineage^+^ cells.

**Fig. S4. Determination of the absence of conventional NK cells in CD45^+^, lineage^-^ population**


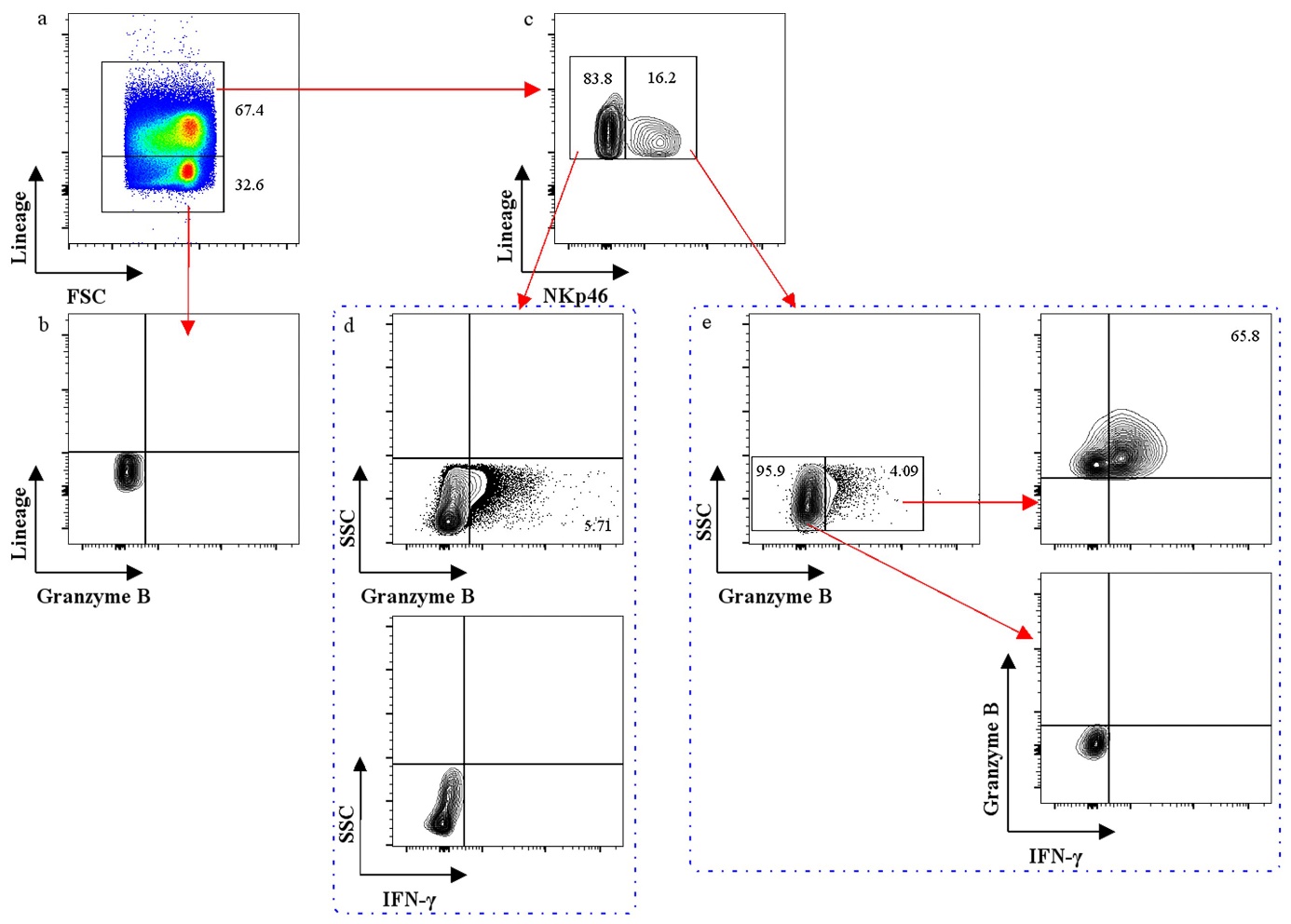


Lung cells from BALB/c mice immunized with FPV-HIV control vaccine were firstly gated on CD45^+^, FSC^low^ and SSC^low^ followed by lineage^-^ and lineage^+^ populations **(a)**, and the granzyme B expression was evaluated on these subsets respectively **(b & c)**. The lineage^+^ cells were further divided into NKp46^+^ and NKp46^-^ populations **(c)**, and their granzyme B and IFN-γ production were evaluated **(d & e)**. Data indicated that granzyme B was only expressed by lineage^+^ cells (NKp46^+^ and NKp46^-^) not lineage^-^ cells **(b)**, and IFN-γ expression was also detected in conventional NK cells, which were determined as lineage^+^ NKp46^+^ Granzyme B^+^. Numbers on FACS plots represent cell percentage.

**Fig. S5. Evaluation of IFN-γ expression by lung conventional NK cells following adjuvanted and unadjuvanted i.n. rFPV immunization**


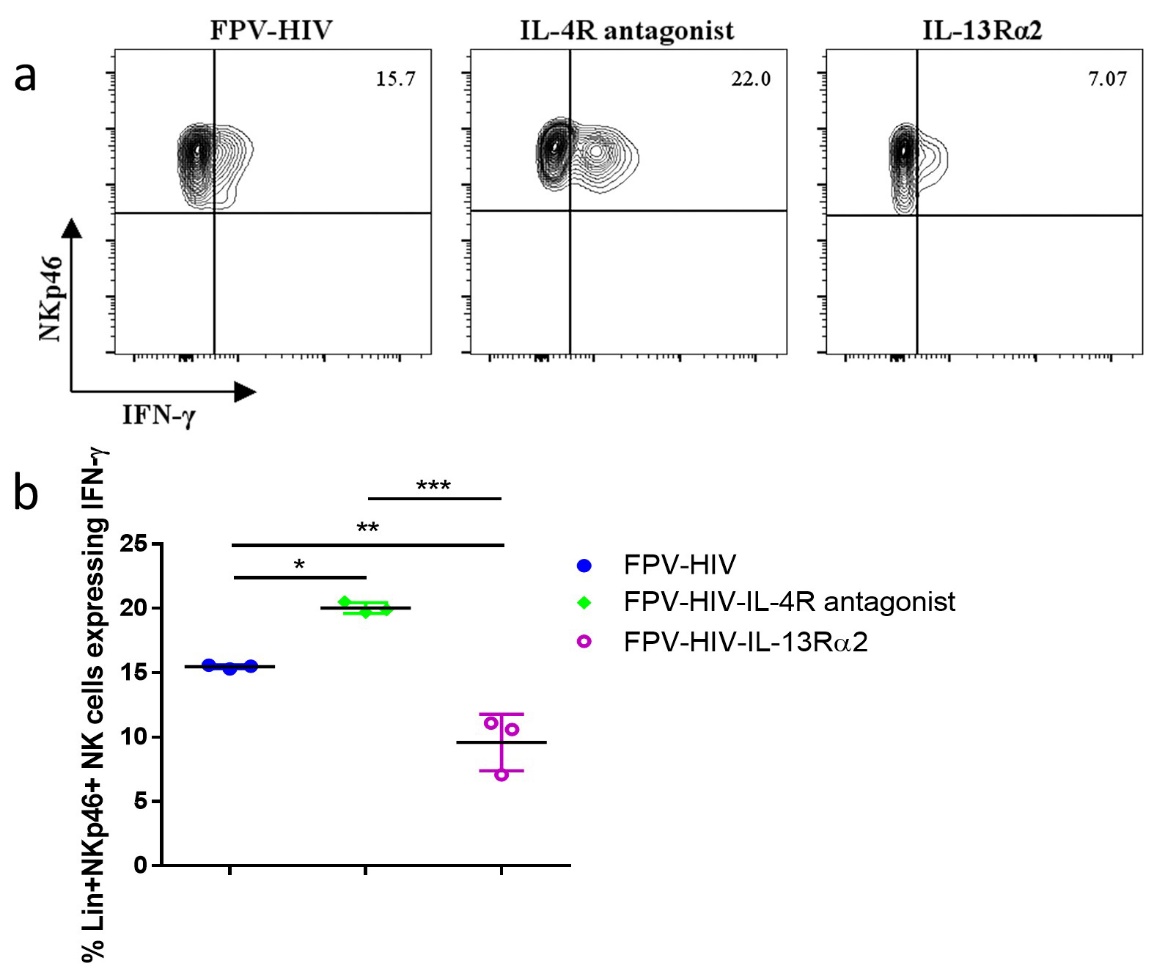


BALB/c mice were immunized intranasally with FPV-HIV, FPV-HIV-IL-4R antagonist adjuvanted, and FPV-HIV-IL-13Rα2 adjuvanted vaccines and IFN-γ expression by conventional NK cells were evaluated at 24h post vaccination in lung. Conventional NK cells were gated as CD45^+^, lineage^+^, and NKp46^+^, and their IFN-γ expression were evaluated using intracellular cytokine staining **(a & b)**. The graphs represent the mean and standard deviation (s.d.). The p-values were calculated using GraphPad Prism software (version 6.05 for Windows). *P<0.05, **p<0.01, ****P<0.0001 (one-way ANOVA). For each time point experiments were repeated minimum three times. Data indicate that 24h post vaccination, the IL-4R antagonist adjuvanted vaccinated group showed elevated IFN-γ expression by the lung lineage^+^ NKp46^+^ (conventional NK cells) subset (~22%) compared to the control unadjuvanted (~15%) or IL-13Rα2 adjuvanted vaccine groups tested (~7%).

**Fig. S6. Staining of lung tissue for different ILC2 subsets**


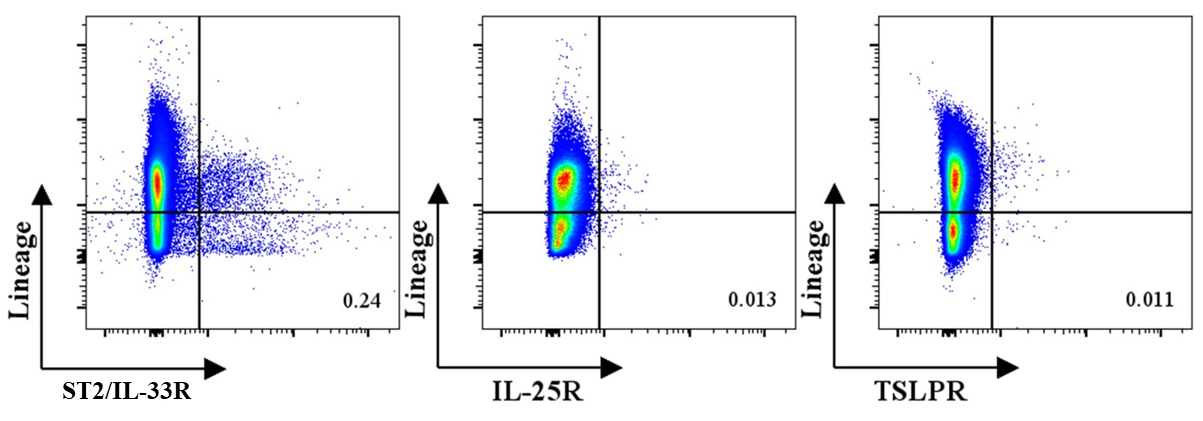


Cells were gated from CD45^+^ and lymphocytes as shown in supplementary Figure 1 and lineage^-^ ST2/IL-33R^+^, lineage^-^ IL-25R^+^, and lineage^-^ TSLPR^+^ lung ILC2 subsets were evaluated 24h post FPV-HIV immunization. Following rFPV immunization, all lung ILC2s were found to be ST2/IL-33R^+^. Numbers on FACS plots represent cell percentage.

**Fig. S7. Evaluation of different ILC2 subsets in naïve mice quadriceps muscle**


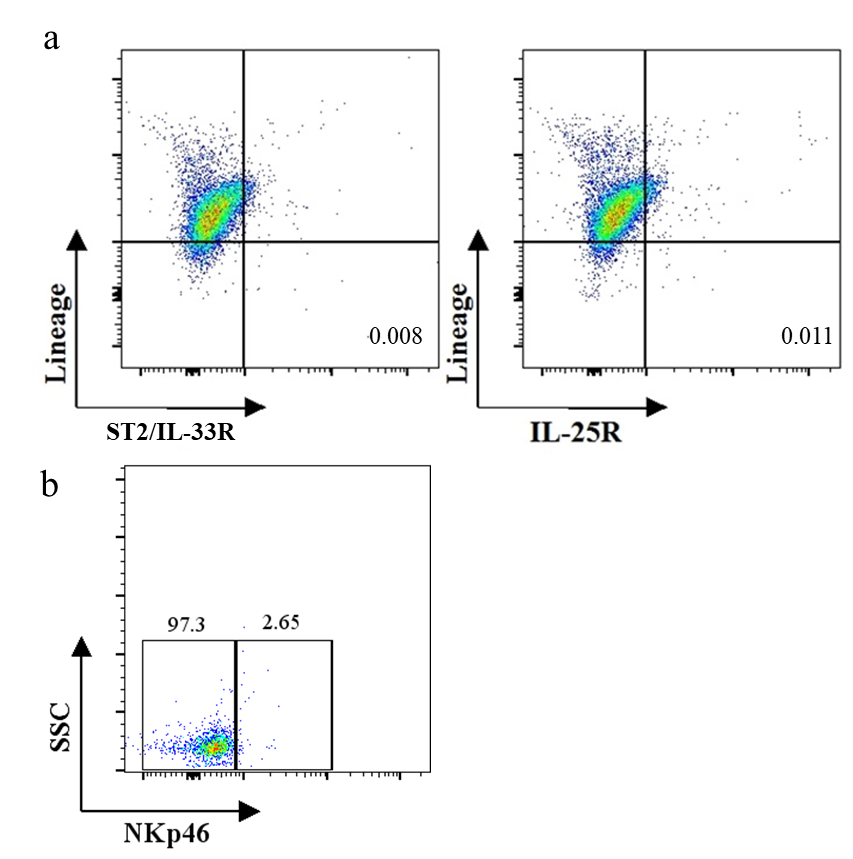


Cells were gated from CD45^+^ and lymphocytes as shown in supplementary Figure 1. No or extremely low ST2/IL-33R^+^ (0.008%) and IL-25R^+^ (0.011%) ILC2 cells were detected in quadriceps muscle in naïve BALB/c mice **(a)**. Very low NKp46+ ILC (18.3%) were detected in quadriceps muscle in naïve BALB/c mice **(b)**.
